# Brain-derived neurotrophic factor from microglia regulates neuronal development in the medial prefrontal cortex and its associated social behavior

**DOI:** 10.21203/rs.3.rs-3094335/v1

**Published:** 2023-06-30

**Authors:** Manabu Makinodan, Takashi Komori, Kazuya Okamura, Minobu Ikehara, Kazuhiko Yamamuro, Nozomi Endo, Kazuki Okumura, Takahira Yamauchi, Daisuke Ikawa, Noriko Ouji-Sageshima, Michihiro Toritsuka, Ryohei Takada, Yoshinori Kayashima, Rio Ishida, Yuki Mori, Kohei Kamikawa, Yuki Noriyama, Yuki Nishi, T Ito, Yasuhiko Saito, Mayumi Nishi, Toshifumi Kishimoto, Kenji Tanaka, Noboru Hiroi

**Affiliations:** Nara Medical University; Nara Medical University School of Medicine; Nara Medical University School of Medicine; Nara Medical University School of Medicine; Nara Medical University School of Medicine; Nara Medical University School of Medicine; Nara Medical University School of Medicine; Nara Medical University School of Medicine; Nara Medical University School of Medicine; Nara Medical University School of Medicine; Nara Medical University School of Medicine; Keio University School of Medicine; Keio University School of Medicine; Keio University School of Medicine; Keio University School of Medicine; Keio University School of Medicine; Keio University School of Medicine; Keio University School of Medicine; Keio University School of Medicine; Keio University School of Medicine; Keio University School of Medicine; Keio University School of Medicine; Keio University School of Medicine; University of Texas Health Science Center at San Antonio

## Abstract

Microglia and brain-derived neurotrophic factor (BDNF) are essential for the neuroplasticity that characterizes critical developmental periods. The experience-dependent development of social behaviors—associated with the medial prefrontal cortex (mPFC)—has a critical period during the juvenile period in mice. However, whether microglia and BDNF affect social development remains unclear. Herein, we aimed to elucidate the effects of microglia-derived BDNF on social behaviors and mPFC development. Mice that underwent social isolation during p21–p35 had increased *Bdnf* in the microglia accompanied by reduced adulthood sociability. Additionally, transgenic mice overexpressing microglia *Bdnf*—regulated using doxycycline at different time points—underwent behavioral, electrophysiological, and gene expression analyses. In these mice, long-term overexpression of microglia BDNF impaired sociability and excessive mPFC inhibitory neuronal circuit activity. However, administration of doxycycline to normalize BDNF from p21 normalized sociability and electrophysiological functions; this was not observed when BDNF was normalized from a later age (p45–p50). To evaluate the possible role of BDNF in human sociability, we analyzed the relationship between adverse childhood experiences and *BDNF* expression in human macrophages, a possible substitute for microglia. Results show that adverse childhood experiences positively correlated with *BDNF* expression in M2 but not M1 macrophages. Thus, microglia BDNF might regulate sociability and mPFC maturation in mice during the juvenile period. Furthermore, childhood experiences in humans may be related to BDNF secretion from macrophages.

## Introduction

Microglia refine synapses and form developing brain circuits in an activity-dependent manner [1, 2]. This process is essential for normal brain development [3]. A limited critical postnatal period of development exists in the sensory cortexes of mice, during which neural network plasticity is transiently enhanced, and the experience-dependent flexible reorganization of neural circuits occurs [4, 5]. Microglia are involved in activity-dependent brain formation processes during brain development [1–3, 6, 7]. Notably, recent studies suggest that microglia may be actively involved in the critical-period plasticity [8–10].

Furthermore, similar to sensory functions, social behaviors develop via experience-dependent brain maturation during a limited postnatal window [11, 12]. For instance, mice that undergo social isolation 21–35 days postnatal (p21–35) later display social impairment that is irreversible by re-socialization [12]. Juvenile social deprivation harms the neural circuits and glial cells of the medial prefrontal cortex (mPFC) [11–18], which is a control center of social behaviors [19]. Previously, we reported the involvement of microglia in juvenile social isolation and future social impairment [13]. Meanwhile, others have implicated microglia in social development [20–22]. Additionally, microglia play a role in the synaptic pruning of the prefrontal cortex [23] and in the time-specific functional maturation of mPFC in rodents [24]. These findings suggest that microglia are involved in mPFC maturation and its associated sociability function in a time-specific manner via activity-dependent neuroplasticity and pruning.

Brain-derived neurotrophic factor (BDNF) contributes to maturing inhibitory interneurons and closing critical periods [25]. Microglia secrete BDNF [26–28], which is relevant for learning-dependent synaptic plasticity in the motor cortex [29] and its effect on inhibitory synaptic transmission in the spinal cord [26]. However, it remains unclear whether microglia-secreted BDNF (MG-BDNF) influences the cortical critical-period plasticity and behavior [8, 30]. In this study, we investigate the time-specific effects of microglia on mPFC biology and social behavior, focusing on MG-BDNF.

## Materials and Methods

### Animals

All study protocols were approved by the Animal Care Committee of Nara Medical University in accordance with the policies established in the National Institutes of Health Guide for the Care and Use of Laboratory Animals. The animals were housed in a temperature- and humidity-controlled animal facility under a standard 12-h light-dark cycle (lights on 08:00–20:00) with *ad libitum* access to food and water throughout the study. C57BL/6J mice isolated from the weaning age (p21) for two weeks were regrouped with age-, sex-, and strain-matched mice at p35 (3–5 mice per cage), labeled juvenile social isolation (j-SI) mice. Control C57BL6/J mice were group-housed following weaning at p21 and labeled Group-Housed (GH) mice. We crossed ionized calcium-binding adapter molecule 1(*Iba1*) promoter driving tetracycline transactivator (*Iba1*-tTA) transgenic mice (line 75) [31] with tetracycline operator-*Bdnf* knock-in homozygous (*Bdnf*^tetO/tetO^) mice [32, 33] and obtained microglia-specific *Bdnf*-overexpressing mice (*Iba1*-tTA::*Bdnf*^tetO/+^ mice) and control mice (*Bdnf*^tetO/+^ mice). Both *Iba1*-tTA and *Bdnf*^tetO/tetO^ mice strains had a mixed C57BL/6J and 129/SvEv background; only F1 male mice were used for all experiments [34]. Genotyping was confirmed as in previous studies [31, 32]. To temporally control *Bdnf* overexpression, F1 mice were fed doxycycline (DOX)-containing chow (DOX 100 mg/kg, CLEA Japan Inc., Tokyo, Japan): from weaning (p21) or adulthood (p45–p50). Groups of mice administered DOX from adulthood were provided chow with DOX ad libitum for at least two weeks before the experiment.

### Statistical analysis

All statistical analyses were performed using Prism v.9 (GraphPad Software Inc., San Diego, CA, USA). When the sample data were normally distributed and had equal variances, as determined using the Shapiro-Wilk test and the subsequent F-test, significant differences between groups were assessed using an unpaired two-tailed Student’s *t*-test. Even if the sample data passed the Shapiro-Wilk test for normality, the nonparametric Mann–Whitney *U*-test was used if the F-test indicated unequal variances. When the sample data significantly differed from normal distribution as assessed by the Shapiro-Wilk test, the nonparametric Mann–Whitney U-test was used. Values with right-skewed distribution were log-transformed for statistical analyses. The Kruskal-Wallis test was used to validate microglia-specific BDNF-overexpressing levels. Two-way ANOVA followed by a Bonferroni post hoc test analyzed the time spent in each zone in the three-chamber social preference test [35]. Two-way repeated measures ANOVA was used to assess the cumulative number of approaches in the augmented reality-based long-term animal behavior observing system. Spearman’s rank correlation coefficient was used to evaluate correlations between *Bdnf* expression in peripheral blood mononuclear cells (PBMC) and microglia in mice, as well as between child abuse and trauma scale (CATS) scores and *BDNF* in macrophages in humans. The false discovery rate-controlling Benjamini–Hochberg procedure was used to control for multiple comparisons [36]. All data were presented as the mean ± standard error of the mean (SEM); pvalues < 0.05 were considered statistically signi cant. The q-values were determined for multiple comparisons, and values < q-value were significant.

Detailed methodological information is provided in the Supplementary materials and methods.

## Results

### Juvenile social isolation (j-SI) mice are socially impaired and have increased MG- Bdnf

**Figure 1:**
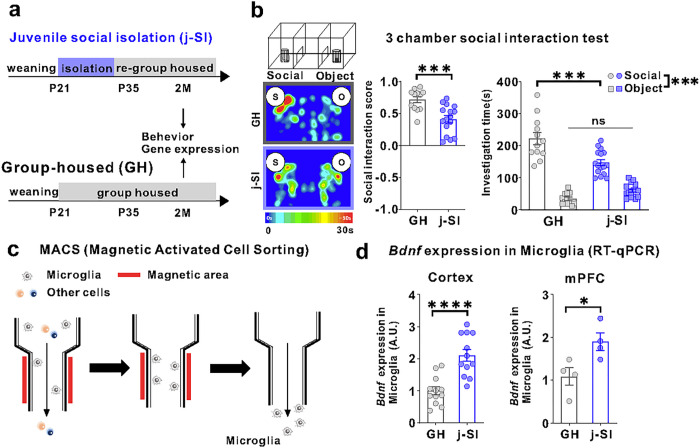
Juvenile social isolation reduces sociability and increases MG-*Bdnf* expression. (a) Schema of the GH and juvenile social isolation (j-SI) mice time series. The experiments were started at P56. (b) Sociability j-SI mice displayed reduced sociability in the three-chamber sociability test compared with GH mice. j-SI mice had a lower social interaction score than GH mice (t_(26)_ = 3.948, p =0.0005, unpaired two-tailed Student’s *t*-test, GH: n = 12, j-SI: n = 16) and spent less time near novel mice (F_1,26_ (interaction) = 17.16, p = 0.0003, two-way ANOVA followed by Bonferroni post hoc test, Bonferroni post hoc analysis of GH S vs. j-SI S, p < 0.0001 GH: n = 12, j-SI: n = 16). S, social; O, object. (c) Image of magnetic-activated cell sorting. (d) (Left) *Bdnf* expression measured using real-time quantitative polymerase chain reaction (RT-qPCR) in microglia recovered from the cortex was higher in the j-SI mice than in the GH mice (t_(22)_ = 4.876, p < 0.0001, unpaired two-tailed Student’s *t*-test, GH: n = 12, j-SI: n = 12). (Right) *Bdnf* expression measured using RT-qPCR in microglia recovered from the mPFC was higher in j-SI than in GH mice (t_(6)_ = 2.818, p=0.0304, unpaired two-tailed Student’s *t*-test, GH: n = 4 (from 16 mice), j-SI: n = 4 (from 16 mice)). The value was log-transformed with a base of 10 because of the right-skewed distribution. Each dot indicates the expression of *Bdnf* in microglia collected from the mPFC of four mice. *p < 0.05, ***p < 0.001, ****p < 0.0001. Data are presented as the mean ± SEM. 2M: two months of age, A.U.: arbitrary unit

Our single housing condition started at P21 and lasted for two weeks, followed by re-grouped housing with age-, sex-, and strain-matched mice (Fig. 1a). While single housing conditions induce varying alterations in the brain and behavioral consequences in mice, depending on the strain, sex, and age [37, 38], our procedure has been shown to induce agitation and defective social behaviors and alter the plasticity in the mPFC [12, 14, 15, 39, 40]. In this study, C57BL/6J male mice isolated during p21–p35 also exhibited impaired sociability in a three-chamber social preference test at two months of age (Fig. 1a, b). In the open field test, no differences in basal activity or anxiety existed between j-SI and GH mice (Supplementary Fig. 1a, b). *Bdnf* mRNA expression in microglia from the cerebral and prefrontal cortexes was higher in j-SI mice than GH mice (Fig. 1c, d). However, no differences were observed in *Bdnf* mRNA expression in the bulk tissues of the cerebral cortex and mPFC between the j-SI and GH mice (Supplementary Fig. 1a, c). These findings indicate that microglial *Bdnf* expression changes are specific to microglia [29]. Moreover, the lack of social stimulation during the juvenile period or subsequent re-socialization increases microglial *Bdnf* expression in the cerebral cortex, including the mPFC.

### Persistently overexpressed MG-BDNF causes social impairment and functional changes in the mPFC pyramidal cells

**Figure 2:**
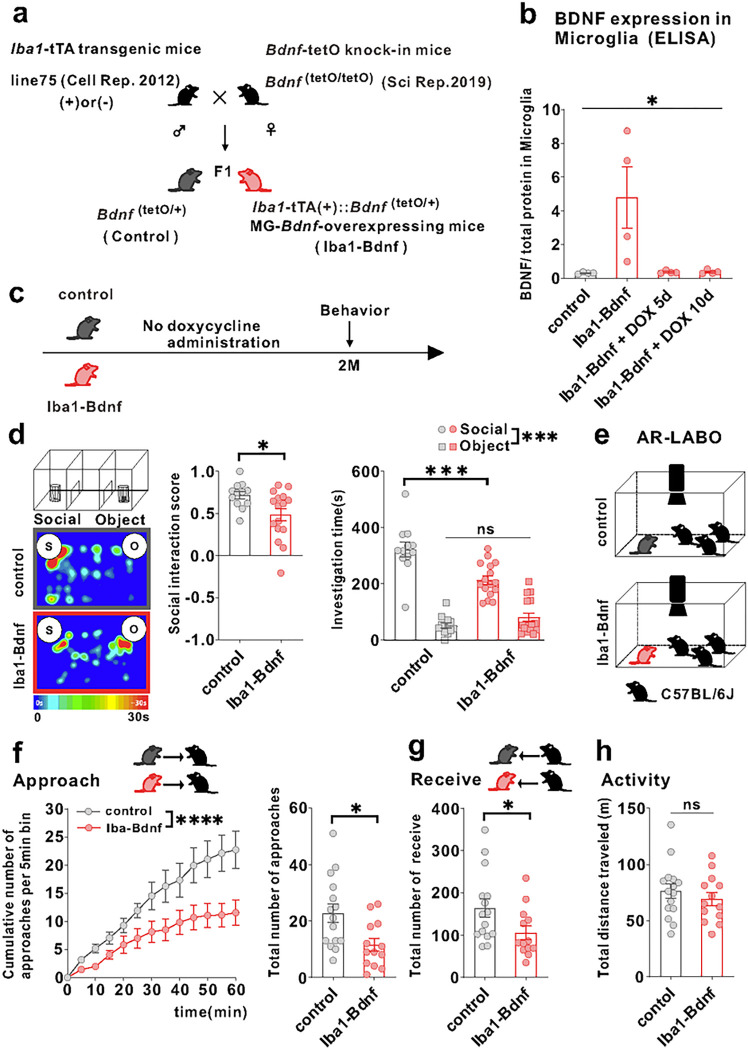
Overexpression of MG-BDNF leads to impaired sociability in adulthood. (a) Diagram of the generation of MG-*Bdnf*-overexpressing mice. The tet-off system was activated in double transgenic mice (F1), which were obtained by crossing *Iba1*-tTA transgenic mice with *Bdnf*-tetO knock-in mice. *Iba1*-tTA(+)::*Bdnf*^(tetO/+)^ mice were the MG-*Bdnf*-overexpressing mice, and *Bdnf*^(tetO/+)^ mice were the control mice. (b) BDNF expression in microglia recovered from the cortex, as measured using ELISA. *Iba1*-tTA(+)::*Bdnf*^(tetO/+)^ mice had higher BDNF expression than *Bdnf*^(tetO/+)^ mice; this was normalized from day 5 using doxycycline (The Kruskal-Wallis test, 9.086 , p= 0.0118; n = 4 per group). (c) Schema of the behavioral experiments without doxycycline. The experiments were started at P61. (d) The *Iba1*-tTA(+)::*Bdnf*^(tetO/+)^ mice were less social in the three-chamber sociability test than the *Bdnf*^(tetO/+)^ mice. The *Iba1*-tTA(+)::*Bdnf*^(tetO/+)^ mice had a lower social interaction score than *Bdnf*^(tetO/+)^ mice (U = 47, p = 0.0226, Mann–Whitney U test, *Bdnf*^(tetO/+)^: n = 12, *Iba1*-tTA(+)::*Bdnf*^(tetO/+)^: n = 16)(left) and spent less time near novel mice (F_1,26_(interaction) = 14.63, p = 0.0007, two-way ANOVA followed by Bonferroni post hoc test; Bonferroni post hoc analysis of *Iba1*-tTA(+)::*Bdnf*^(tetO/+)^ S vs. *Bdnf*^(tetO/+)^ S, p = 0.0001, *Bdnf*^(tetO/+)^: n = 12, *Iba1*-tTA(+)::*Bdnf*^(tetO/+)^: n = 16)(right). S, social; O, object (e) Schematic of the Augmented Reality-based Long-term Animal Behavior Observing system (AR-LABO). Three novel C57BL/6J mice were placed with a target mouse and observed for 1 hr. (f) (Left) The cumulative number of approaches per 5-min bin for *Bdnf*^(tetO/+)^ and *Iba1*-tTA(+)::*Bdnf*^(tetO/+)^ mice. *Iba1*-tTA(+)::*Bdnf*^(tetO/+)^ mice accumulates significantly less cumulative approaches to other mice than *Bdnf*^(tetO/+)^ mice (two-way RM ANOVA, phenotype (control or Iba1-BDNF) × time (5 min bin) interaction F_(12,312)_ = 3.926, p <0.0001; effect of phenotype F_(1,26)_ = 6.670, p = 0.0158; effect of time F_(12,312)_ = 39.31, p <0.0001 , *Bdnf*^(tetO/+)^: n =15, *Iba1*-tTA(+)::*Bdnf*^(tetO/+)^: n = 13). (Right) *Iba1*-tTA(+)::*Bdnf*^(tetO/+)^ mice approached other mice less than the *Bdnf*^(tetO/+)^ mice (t_(26)_ = 2.717, p = 0.0116, unpaired two-tailed Student’s *t*-test, *Bdnf*^(tetO/+)^: n =15, Iba1-tTA(+)::*Bdnf*^(tetO/+)^: n = 13). (g) The Iba1-tTA(+)::*Bdnf*^(tetO/+)^ mice were less approached by other mice than *Bdnf*^(tetO/+)^ mice (t_(26)_ = 2.120, p = 0.0437, unpaired two-tailed Student’s *t*-test, *Bdnf*^(tetO/+)^: n = 15, *Iba1*-tTA(+)::*Bdnf*^(tetO/+)^: n = 13). (h) No differences in activity were observed during AR-LABO between the *Iba1*-tTA(+)::*Bdnf*^(tetO/+)^ and *Bdnf*^(tetO/+)^ mice (t_(26)_ = 0.8059, p = 0.4276, unpaired two-tailed Student’s *t*-test, *Bdnf*^(tetO/+)^: n = 15, *Iba1*-tTA(+)::*Bdnf*^(tetO/+)^: n = 13). *p < 0.05, ***p < 0.001. Data are presented as the mean ± SEM. 2M: two months of age, control: *Bdnf*^(tetO/+)^ mice, Iba1-BDNF: *Iba1*-tTA(+)::*Bdnf*^(tetO/+)^ mice

To recapitulate the microglia-specific and sustained *Bdnf* overexpression observed in j-SI and examine the causal relationship between microglia-specific *Bdnf* gene upregulation and social impairment, we exploited a tet-off system [41] to induce microglia-specific *Bdnf* mRNA expression (Fig. 2a). MG-BDNF overexpression was con rmed in *Iba1*-tTA::*Bdnf*^(tetO/+)^ mice without DOX and MG-BDNF overexpression in *Iba1*-tTA::*Bdnf*^(tetO/+)^ mice was normalized from day 5 after orally administering DOX (Fig. 2b). *Bdnf* mRNA expressions in the bulk tissues of the cerebral cortex and mPFC did not differ between the *Iba1*-tTA::*Bdnf*^(tetO/+)^ and control mice, suggesting that genetically-induced MG-*Bdnf* overexpression in *Iba1*-tTA::*Bdnf*^*(*tetO/+)^ mice does not significantly alter total *Bdnf* mRNA expression levels in the cerebral cortex or mPFC (Supplementary Fig. 2a, b).

Next, we evaluated whether persistent MG-BDNF overexpression impairs social behavior at two months of age such as j-SI mice using a three-chamber social preference test (Fig. 2c). The *Iba1*-tTA::*Bdnf*^(tetO/+)^ mice had a lower social interaction score than the control mice and spent less time around the novel mice (Fig. 2d). In contrast, no differences in locomotor activity or anxiety existed between the *Iba1*-tTA::*Bdnf*^(tetO/+)^ and control mice (Supplementary Fig. 2a, c). However, considering that the three-chamber apparatus has certain interpretative issues [42] and that social behavior through a barrier has different molecular substrates than true physical social interactions [43], we further assessed real social behavior in a naturalistic environment among multiple animals using the augmented reality-based long-term animal behavior observing system (AR-LABO). This system is an improved version of our previous system that can determine the position of each mouse under social housing conditions [44–46]. The subject mice and three age-matched male C57BL/6J mice that had never been cohoused with each other were placed in a new cage at two months of age, and their movements were observed for 1 h (Fig. 2e). Compared to the control, the *Iba1*-tTA::*Bdnf*^(tetO/+)^ mice made fewer approaches to other mice (Fig. 2f). They were also less often approached by other mice (Fig. 2g). No difference in total activity existed between the *Iba1*-tTA::*Bdnf*^(tetO/+)^ and control mice during the 1-h recording time (Fig. 2h). These results suggest that sustained MG-BDNF overexpression induces social deficits in adulthood.

**Figure 3:**
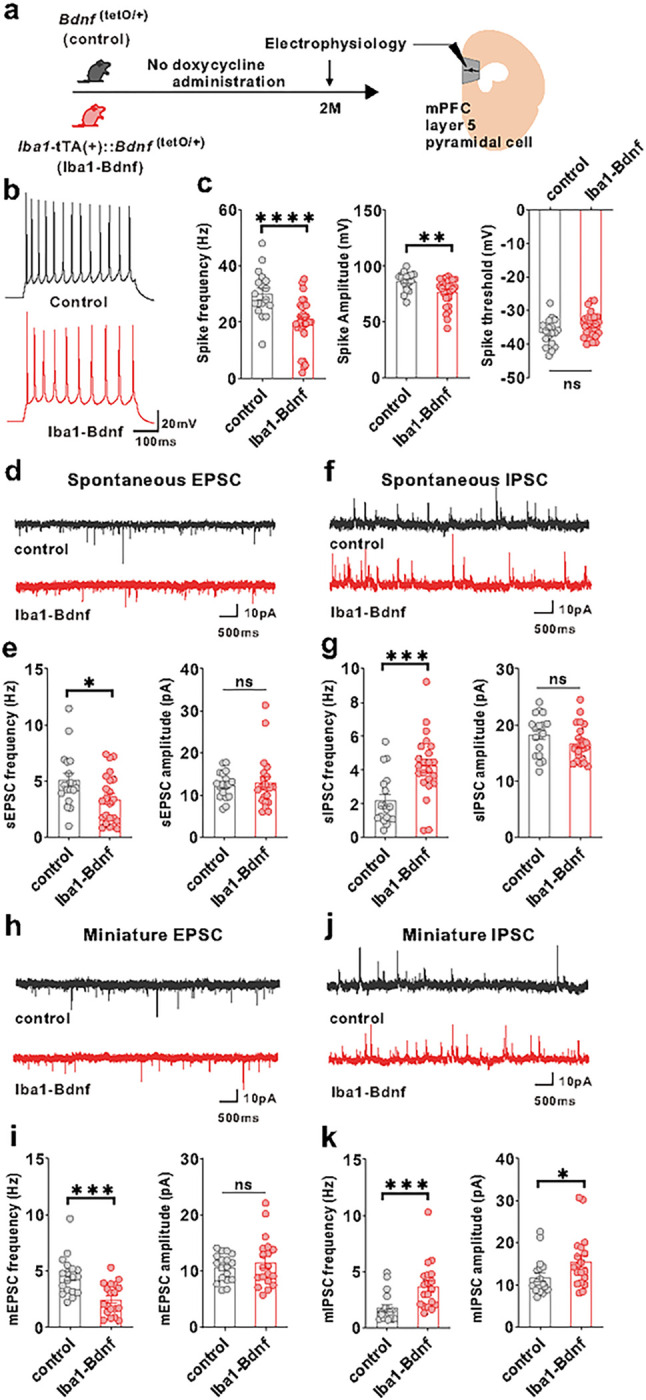
MG-BDNF overexpression affects the inhibitory synaptic inputs to mPFC pyramidal cells and the excitability of such cells. (a) Electrophysiological analysis of mPFC layer V pyramidal cells in adulthood without doxycycline administration. The experiments were started at P62. (b) Representative traces recorded from mPFC pyramidal cells at 200-pA injection. (c) *Iba1*-tTA(+)::*Bdnf*^(tetO/+)^ mice had lower spike frequency (U = 93.50, p < 0.0001, Mann–Whitney U test) (left) and amplitude (U = 131, p = 0.0026, Mann–Whitney U test) (middle) than *Bdnf*^(tetO/+)^ mice at 200-pA injection. No significant differences existed in spike threshold (t_(46)_ =1.997, p = 0.0518, unpaired two-tailed Student’s t-test) (right). (n = 18 cells from three biologically independent *Bdnf*^(tetO/+)^ mice, n = 30 cells from four biologically independent *Iba1*-tTA(+)::*Bdnf*^(tetO/+)^ mice). (d) Representative traces of spontaneous EPSCs. (e) (Left) The *Iba1*-tTA(+)::*Bdnf*^(tetO/+)^ mice had lower sEPSC frequency than *Bdnf*^(tetO/+)^ mice (U = 130, p = 0.0436, Mann–Whitney U test). (Right) No significant difference existed in sEPSC amplitude (U = 185, p = 0.5761, Mann–Whitney U test). (f) Representative traces of spontaneous IPSCs. (g) (Left) *Iba1*-tTA(+)::*Bdnf*^(tetO/+)^ mice showed increased sIPSC frequency compared with *Bdnf*^(tetO/+)^ mice (U = 85, p = 0.0010, Mann–Whitney U test). (Right) There was no significant difference in sIPSC amplitude (t_(39)_ = 1.440, p = 0.1579, unpaired two-tailed Student’s t-test). (e, g) n = 18 cells from three biologically independent *Bdnf*^(tetO/+)^ mice, n = 23 cells from four biologically independent *Iba1*-tTA(+)::*Bdnf*^(tetO/+)^ mice. (h) Representative traces of miniature EPSCs. (i) (Left) *Iba1*-tTA(+)::*Bdnf*^*(*tetO/+)^ mice had lower mEPSC frequency than *Bdnf*^(tetO/+)^ mice. (t_(35)_ = 3.949, p = 0.0004, unpaired two-tailed Student’s t-test). (Right) There was no significant difference in mEPSC amplitude (U = 158, p = 0.7074, Mann–Whitney U test). (j) Representative traces of miniature IPSCs. (k) The *Iba1*-tTA(+)::*Bdnf*^(tetO/+)^ mice had increased mIPSC frequency (U = 53, p = 0.0002, Mann–Whitney U test)(left) and amplitude (U = 101, p = 0.0335, Mann–Whitney U test)(right) than the *Bdnf*^(tetI/+)^ mice. (i, k) n = 18 cells from three biologically independent *Bdnf*^(tetO/+)^ mice, n =19 cells from three biologically independent *Iba1*-tTA(+)::*Bdnf*^(tetO/+)^ mice. *p < 0.05, **p < 0.01, ***p < 0.001, ****p < 0.0001. Data are presented as the mean ± SEM. 2M: two months of age, control: *Bdnf*^(tetO/+)^ mice, Iba1-BDNF: *Iba1*-tTA(+)::*Bdnf*^(tetO/+)^ mice

To address whether artificial BDNF overexpression causes mPFC dysfunction, we examined the mPFC layer V pyramidal cells using whole-cell patch-clamp recordings at two months of age (Fig. 3a). Compared with the adult control mice, excitability was significantly reduced in the adult *Iba1*-tTA::*Bdnf*^(tetO/+)^ mice in terms of spike frequency and amplitude (Fig. 3b, c). In contrast, no significant differences existed in the spike thresholds (Fig. 3c). Spontaneous excitatory postsynaptic current (sEPSC) frequency was significantly lower (Fig. 3d, e), and spontaneous inhibitory postsynaptic current (sIPSC) frequency was significantly higher (Fig. 3f, g) in the adult *Iba1*-tTA::*Bdnf*^(tetO/+)^ mice compared with the control mice. In contrast, sEPSC and sIPSC amplitudes did not significantly differ (Fig. 3e, g). Furthermore, in *Iba1*-tTA::*Bdnf*^(tetO/+)^ mice, miniature EPSC (mEPSC) frequency was significantly lower (Fig. 3h, i), and miniature IPSC (mIPSC) frequency and amplitude were significantly higher (Fig. 3j, k) than in the control mice (Fig. 3k). These results suggest that a sustained increase in MG-BDNF from early childhood may decrease the excitability of mPFC layer V pyramidal cells. It also increases the number of inhibitory synaptic inputs and decreases the excitatory synaptic inputs in such cells, enhancing the activity of inhibitory neuronal circuits. In summary, our results demonstrated that overexpressed MG-BDNF was sufficient to induce mPFC dysfunction and social impairment.

### MG-BDNF affects the complement system in the mPFC

**Figure 4:**
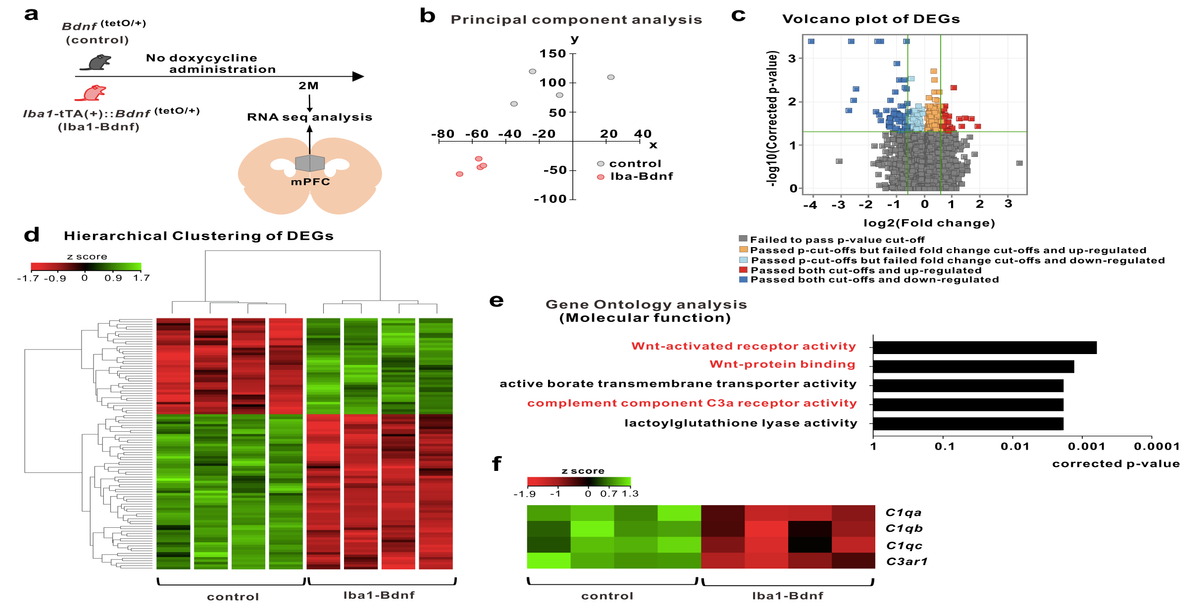
MG-BDNF overexpression affects the complement system. (a) RNA-seq analysis of the mPFC in adult *Bdnf*^(tetO/+)^ (n = 4) and *Iba1*-tTA(+)::*Bdnf*^(tetO/+)^ (n = 4) mice without doxycycline. The experiments was started at P64. (b) Principal component analysis revealing gene expression differences between the *Bdnf*^(tetO/+)^ and *Iba1*-tTA(+)::*Bdnf*^(tetO/+)^ mice. (c) Volcano plot of differentially expressed genes (DEGs). The thresholds are log_2_ fold change > 1.5 and p < 0.05. (d) Heatmap and hierarchical clustering of DEGs between *Bdnf*^(tetO/+)^ and *Iba1*-tTA(+)::*Bdnf*^(tetO/+)^ mice. The thresholds are log_2_ fold change > 1.5 and p < 0.05. Gene expression levels are indicated in the heatmap by the Z-scores in the legend. (e) The Gene Ontology analysis of down-regulated DEGs in the *Iba1*-tTA(+)::Bdnf^(tetO/+)^ mice compared with *Bdnf*^(tetO/+)^ mice suggested the involvement of the Wnt signaling pathway (Wnt-activated receptor pathway, p = 0.0006; Wnt-protein binding, p = 0.0013) and complement component C3a receptor activity (p = 0.0018) in molecular function. (f) Differences in expression levels of selected complement genes in *Bdnf*^(tetO/+)^ and *Iba1*-tTA(+)::*Bdnf*^(tetO/+)^ mice in RNA-Seq analysis. Gene expression levels are presented in the heatmap by the Z-scores in the legend. 2M: two months of age, control: *Bdnf*^(tetO/+)^ mice, Iba1-BDNF: Iba1-tTA(+)::*Bdnf*^(tetO/+)^ mice

RNA-sequencing and principal component analysis revealed that MG-BDNF overexpression during the juvenile period altered mPFC distribution at two months of age (Fig. 4a, b). Compared with control mice, 63 genes were downregulated, and 39 were upregulated in the adult *Iba1*-tTA::*Bdnf*^(tetO/+)^ mice (Fig. 4c, d). Gene Ontology molecular function analysis using differentially expressed genes downregulated in *Iba1*-tTA::*Bdnf*^(tetO/+)^ mice revealed that sustained MG-BDNF overexpression altered gene expression related to Wnt-activated receptor activity (corrected p-value < 0.0001), Wnt-protein binding (corrected p-value < 0.0001), active borate transmembrane transporter activity (corrected p-value = 0.0018), and complement C3a receptor activity (corrected p-value = 0.0018; Fig. 4e). Heatmaps of Wnt signaling- and complement system-related gene expression were indicated with Z-scores, referencing the Kyoto Encyclopedia of Genes and Genomes pathways [47] (Supplementary Fig. 3 and Fig. 4f). This heat mapping of *C1qa*, *C1qb*, *C1qc*, and *C3ar1* expression using Z-scores suggested reduced function of this complement cascade beginning with *C1q* in *Iba1*-tTA::*Bdnf*^(tetO/+)^ mice. In particular, the *Iba1*-tTA::*Bdnf*^(tetO/+)^ mice had significantly decreased *C1qa* (corrected p-value = 0.0241) and *C3ar1* expression (corrected p-value = 0.0288). These results suggest that MG-BDNF overexpression decreases the complement cascade functional status in the mPFC.

### Normalizing MG-BDNF overexpression during the juvenile period rescues impaired sociability and mPFC pyramidal cell dysfunction in adulthood

**Figure 5:**
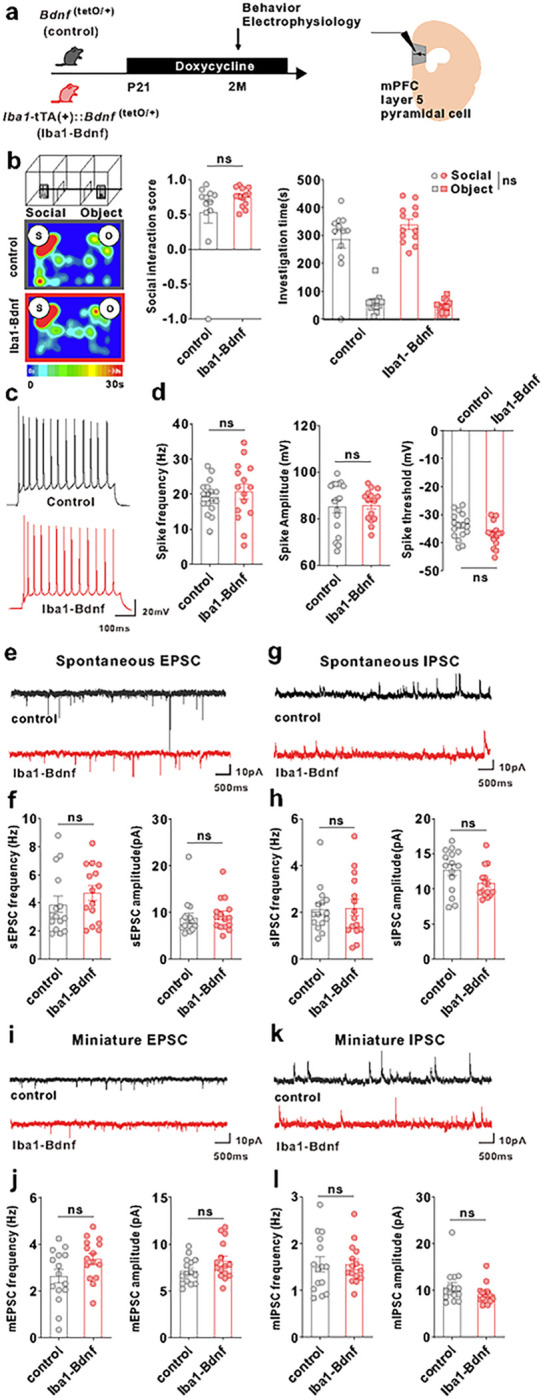
Normalizing MG-BDNF during the juvenile period does not impair sociability or abnormal inhibitory inputs in the mPFC. (a–l) The *Iba1*-tTA(+)::*Bdnf*^(tetO/+)^ mice were administered doxycycline from p21 at weaning to normalize MG-BDNF. *Bdnf*^(tetO/+)^ mice were also administered doxycycline from p21 as the control. Behavioral and electrophysiological experiments were started at p61. (b) Normalizing MG-BDNF from p21 did not reduce sociability in adult *Iba1*-tTA(+)::*Bdnf*^(tetO/+)^ mice in the three-chamber social test. Both groups had no differences in the social interaction score (U = 57, p = 0.2701, Mann–Whitney U test, *Bdnf*^(tetO/+)^: n = 12, *Iba1*-tTA(+)::*Bdnf*^(tetO/+)^: n = 13)(left) or social investigation time (F_1,23_(interaction) = 2.626, p = 0.1187, two-way ANOVA, *Bdnf*^(tetO/+)^: n = 12, *Iba1*-tTA(+)::*Bdnf*^(tetO/+)^: n = 13)(right). S, social; O, object. (c) Representative traces recorded from mPFC pyramidal cells at 200-pA injection. (d) No differences existed between *Bdnf*^(tetO/+)^ and *Iba1*-tTA(+)::*Bdnf*^(tetO/+)^ mice treated with doxycycline from p21 in the spike frequency (U = 110, p = 0.5192, Mann–Whitney U test)(left), spike amplitude (U = 120, p = 0.7944, Mann–Whitney U test)(middle), or threshold (t_(30)_ = 2.026, p = 0.0517, unpaired two-tailed Student’s *t*-test)(right) at 200-pA injection (n = 17 cells from three biologically independent *Bdnf*^(tetO/+)^ mice, n = 15 cells from four biologically independent *Iba1*-tTA(+)::*Bdnf*^(tetO/+)^ mice). (e) Representative traces of spontaneous EPSCs. (f) No differences were observed between *Bdnf*^(tetO/+)^ and *Iba1*-tTA(+)::*Bdnf*^(tetO/+)^ mice treated with doxycycline from p21 in sEPSC frequency (U = 85, p = 0.2671, Mann–Whitney U test)(left) or amplitude (U = 89, p = 0.3453, Mann–Whitney U test)(right). (g) Representative traces of spontaneous IPSCs. (h) Normalizing MG-BDNF from p21 did not increase sIPSC frequency (U = 107, p = 0.8381, Mann–Whitney U test)(left) or amplitude (U = 69, p = 0.0742, Mann–Whitney U test)(right) in *Iba1*-tTA(+)::Bdnf^(tetO/+)^ mice. (f, h) n = 15 cells from ve biologically independent *Bdnf*^(tetO/+)^ mice, n = 15 cells from three biologically independent *Iba1*-tTA(+)::*Bdnf*^(tetO/+)^ mice. (i) Representative traces of miniature EPSCs. (j) No differences existed between *Bdnf*^(tetO/+)^ and *Iba1*-tTA(+)::*Bdnf*^(tetO/+)^ mice treated with doxycycline from p21 in mEPSC frequency (t_(28)_ = 1.988, p = 0.0566, unpaired two-tailed Student’s t-test) (left) or amplitude (t_(28)_ = 1.729, p = 0.0948, unpaired two-tailed Student’s t-test)(right). (k) Representative traces of miniature IPSCs. (l) Normalizing MG-BDNF from p21 did not increase mIPSC frequency (t_(28)_ = 0.01783, p = 0.9859, unpaired two-tailed Student’s *t*-test)(left) or amplitude (U = 76, p = 0.1370, Mann–Whitney U test)(right) in *Iba1*-tTA(+)::*Bdnf*^(tetO/+)^ mice. (j, l) n = 15 cells from five biologically independent Bdnf^(tetO/+)^ mice, n = 15 cells from three biologically independent *Iba1*-tTA(+)::*Bdnf*^(tetO/+)^ mice. Data are presented as the mean ± SEM. 2M: two months of age, control: *Bdnf*^(tetO/+)^ mice, Iba1-BDNF: *Iba1*-tTA(+)::*Bdnf*^(tetO/+)^ mice.

Given that BDNF is known to be associated with the critical period of experience-dependent neural plasticity [25], we manipulated MG-BDNF overexpression by administering DOX at different time points and measured the social behaviors and electrophysiological properties of the mPFC pyramidal neurons in adulthood to investigate whether MG-BDNF regulates social behavior and mPFC function in a time-specific manner. First, MG-BDNF overexpression during the juvenile period was suppressed by oral DOX administration from p21 in the *Iba1*-tTA::*Bdnf*^(tetO/+)^. Similarly, the control mice were also fed DOX from p21 (Fig. 5a). In the three-chamber social preference test, no difference was detected in the social interaction score or time spent around novel mice between the adult *Iba1*-tTA::*Bdnf*^(tetO/+)^ and control mice at two months of age (Fig. 5b), indicating that the suppressing MG-BDNF overexpression from the juvenile period normalized impaired social behavior in adulthood. No significant differences existed in locomotion or anxiety in the open field test (Supplementary Fig. 4). Regarding neuronal function, differences were not observed in spike frequency or amplitude of the excitability of the mPFC layer V pyramidal cells (Fig. 5c, d). Similar results were observed in the sEPSC and mEPSC frequencies (Fig. 5e, f, i, and j). In addition, the sIPSC or mIPSC frequency was comparable between the *Iba1*-tTA::*Bdnf*^(tetO/+)^ and control mice (Fig. 5g, h, k, l). These results suggest that normalizing MG-BDNF expression in *Iba1*-tTA::*Bdnf*^(tetO/+)^ from the juvenile period leads to comparable excitability of mPFC layer V pyramidal cells and its inhibitory inputs to the control mice in adulthood.

Next, we assessed the effect of delayed normalization of MG-BDNF overexpression after p45 on social behavior and mPFC layer V pyramidal cell function. DOX was orally administered to the *Iba1*-tTA::*Bdnf*^(tetO/+)^ and control mice from p45–p50 (Supplementary Fig. 5a). The *Iba1*-tTA::*Bdnf*^(tetO/+)^ and control mice exhibited comparable social interaction scores and time spent around the novel mice at two months of age (Supplementary Fig. 5b). Similarly, no differences existed in locomotion or anxiety during the open field test (Supplementary Fig. 5c). In contrast, normalizing MG-BDNF during adulthood did not improve the electrophysiological abnormalities of mPFC layer V pyramidal cells in the *Iba1*-tTA::*Bdnf*^(tetO/+)^ mice at two months of age. The spike frequency of excitability remained significantly reduced (Supplementary Fig. 5d). In addition, sIPSC and mIPSC frequencies remained significantly increased in the *Iba1*-tTA::*Bdnf*^(tetO/+)^ mice compared with control mice (Supplementary Fig. 5f, h). Furthermore, the sIPSC amplitude was significantly reduced in the *Iba1*-tTA::*Bdnf*^(tetO/+)^ mice compared with control mice (Supplementary Fig. 5f). No significant differences existed in the frequency or amplitude of sEPSCs or mEPSCs (Supplementary Fig. 5e, g). These results suggest that MG-BDNF overexpression during the juvenile period may be critical for forming inhibitory synapses in the mPFC. However, even the MG-BDNF intervention during adulthood can improve social behavior.

### BDNF expression in human M2 macrophages correlates with childhood experiences

Given the significant correlation in *Bdnf* expression between microglia from the brain and peripheral blood mononuclear cells in mice (Supplementary Fig. 6a), we measured *BDNF* expression in human peripheral macrophages, which share properties with microglia [48–50], following the juvenile experience-dependent increase of MG-*Bdnf* in mice. CD14-positive monocytes were collected from the peripheral blood of participants and differentiated into M1/M2 macrophages; subsequently, *BDNF* mRNA expression was measured (Supplementary Fig. 6b). The Japanese version of the CATS was used to assess adverse childhood experiences [51, 52]. A positive correlation existed between the total CATS scores and *BDNF* expression in M2 macrophages. In the CATS sub-items, neglect and punishment, among other variables, positively correlated with *BDNF* expression in M2 macrophages (Table 1). In contrast, no significant correlations existed between *BDNF* expression in M1 macrophages and the CATS total or sub-item scores (Table 1). These results indicate that adverse childhood experiences may increase *BDNF* expression in M2 macrophages, even in humans.

## Discussion

Little is known concerning the microglia’s influence on mPFC development and its function, such as social behavior [53]. As such, microglia are receiving significantly more attention in psychiatric research [54, 55]. In particular, understanding microglia’s role in the mPFC circuit formation is essential due to the critical implication of the mPFC in the pathobiology of neuropsychiatric disorders [53, 56–58]. In this study, we first demonstrated that j-SI mice during p21–p35 had increased MG-*Bdnf* expression and reduced sociability, consistent with previous studies [12, 14]. Next, we investigated the impact of these microglial changes on social behavior and mPFC function using MG-BDNF-overexpressing mice. Sustained MG-BDNF overexpression resulted in impaired social behavior, a reduced ring capacity of mPFC layer V pyramidal cells, and reduced excitatory inputs and enhanced inhibitory inputs to mPFC layer V pyramidal cells, implying an altered excitatory/inhibitory balance. Notably, the post-weaning normalization of MG-BDNF (from p21) ameliorated the impairment of social behaviors and the ring and abnormal excitatory/inhibitory balance in mPFC layer V pyramidal cells. These results suggest that MG-BDNF during the juvenile period is crucial to developing social behaviors and mPFC function. In contrast, when MG-BDNF was normalized from adulthood (p45–p50), the ring and excitatory/inhibitory balance of mPFC layer V pyramidal cells remained abnormal. These findings indicate that MG-BDNF has a critical window of effects on social behavior and mPFC function. The mPFC complement system might be implicated in a possible underlying mechanism, consistent with previous findings that microglial experience-dependent synaptic pruning depends on its related complement system [1, 2, 7].

We used *Bdnf*^*t*etO/+^ mice as the control group and *Iba1*-tTA::*Bdnf*^tetO/+^ mice as the experimental group. Our *Bdnf*^tetO/+^ mice were derived from ES cells of 129/SvEv mice for homologous recombination and backcrossed to C57BL/6J mice for more than five generations. While *Iba1*-tTA mice were originally developed in fertilized eggs of C57BL/6J, they were maintained as breeders with our *Bdnf*^tetO/+^ mice. Accordingly, the alleles near the transgene *Iba1*-tTA are expected to be enriched with those of C57BL/6J and alleles in the rest of the genome contained randomly mixed 129/SvEv and C57BL/6J alleles originating from *Bdnf*^tetO/+^ mice. Thus, the expected impacts of a systematic genetic background bias between the control and experimental groups are minimized, as the alleles near *Iba1*-tTA transgene are those of C57BL/6J in both control and experimental groups and the rest of the genome contained a random mixture of 129/SvEv and C57BL/6J alleles; the mixed genetic backgrounds of *Bdnf*^tetO/+^ mice were present in both the control and experimental groups [34]. Moreover, if the phenotypes reflected genetic background differences between control and experimental groups instead of or in addition to MG-BDNF overexpression, some phenotypic differences between the two groups should have remained after normalizing MG-BDNF levels; no phenotypic difference was seen in social behavior (Fig. 5b) or electrophysiological recordings (Fig. 5c–l).

Recently, Schalbetter et al. reported that microglia affect mPFC function and its relative cognition in a time-specific manner [24]. Although microglia are reportedly related to social behavior [20–22], no study has examined the relationship between sociability and time-specific development of the mPFC with microglia. The j-SI mouse with robust impairment of social behavior is a potential model for human neglect [11, 12]; however, it also makes it possible to elucidate the mechanism of social circuit formation in a limited social experience-dependent window, similar to that of sensory deprivation [59]. Social experience deprivation during the juvenile period (p21–35) has been suggested to affect the excitatory/inhibitory balance in mPFC [16–18], mPFC–pPVT neural circuits [14], and glial cells, such as oligodendrocytes [12] and microglia [13], all of which may be responsible for reduced sociability in these mice. Following the current finding that juvenile social experience deprivation elevates MG-BDNF expression in mPFC, a novel mechanism could elucidate the experience-dependent development of social behaviors. In mice overexpressing MG-BDNF, we demonstrated that higher MG-BDNF expression reduced social behaviors, suggesting that MG-BDNF may be critical in developing social abilities. In addition to a robust social assessment, i.e., the three-chamber social test, we also applied the AR-LABO, in which multiple mice were simultaneously traced under free-moving conditions to confirm their social behavior. MG-BDNF-overexpressing mice exhibited fewer approaches to other mice and received fewer approaches from others. This might be due to MG-BDNF-overexpressing mice emitting lower levels of chemical communication, such as ultrasound, urine, and pheromones [60, 61].

Furthermore, MG-BDNF modulates the excitability and excitatory/inhibitory input of mPFC layer V pyramidal cells in a limited window from the juvenile period (p21) to adulthood (p45–50). This is consistent with the critical period for social ability acquisition in mice, which is from p21 to p35 [12]. In contrast, normalizing MG-BDNF during adulthood did not improve the excitability and excitatory/inhibitory balance of mPFC layer V pyramidal cells, although impaired social behavior was ameliorated. MG-BDNF is implicated in learning-dependent neural plasticity [29] and may modify social circuit formation in brain regions other than the mPFC, even in adulthood [62]. Previous studies have also reported that microglia are related to mPFC circuit formation and cognitive maturation in adolescence [24]; thus, normalizing MG-BDNF after p45–50 may be sufficient to restore sociability.

The excitability of layer V pyramidal cells in the mPFC of MG-BDNF-overexpressing mice is similar to that observed in j-SI mice [16, 18]. This reduction in the excitability of layer V mPFC neurons may be associated with the hypoactivity of mPFC neurons that project subcortically to regulate social behavior [14], leading to reduced sociability. The relationship between MG-BDNF and the development and maturation of inhibitory neuronal circuits is poorly understood; however, enhancing inhibitory neuronal circuits, as in this study, is likely consistent with a known function of BDNF: promoting the formation and maintenance of inhibitory neural synapses during brain development [25, 63–65]. Particularly, BDNF regulates the critical visual cortex period, and overexpressed BDNF leads to the premature maturation of inhibitory neural circuits, leading to early closure of the critical visual cortex period [25]. Juvenile PFC development strengthens inhibitory neurotransmission within the brain, altering the excitatory/inhibitory balance [66, 67], which is implicated in the social function of the mPFC [19]. Overexpressed MG-BDNF might similarly close the critical social development window, disrupting mPFC development and reducing sociability by strengthening inhibitory neural circuits. In this study, normalizing MG-BDNF from adulthood (p45–p50) did not ameliorate the enhanced inhibitory neuronal circuitry. In the rodent neocortex, inhibitory synapse formation primarily occurs postnatally and rapidly (before adolescence) reaches adult-like inhibitory synapse density [68, 69]. The time course of rodent inhibitory synapse functional maturation is similar to that of inhibitory synapse formation. Specifically, IPSC frequency becomes prominent postnatally and displays adult-like properties before adolescence [70]. Enhancing inhibitory neuronal circuits via overexpressed MG-BDNF may increase the density and function of inhibitory synapses. Previous studies have also revealed increased inhibitory inputs in mPFC layer V pyramidal cells in j-SI mice and other abnormalities in inhibitory interneuron functions in the mPFC [11, 15, 17, 18]. Abnormalities in inhibitory circuits induced by juvenile isolation and changes in MG-BDNF expression might be a potential mechanism for the experience-dependent impairment of social development.

In this study, we performed RNA-seq of the mPFC; our findings suggested the involvement of the complement system as a mechanism of MG-BDNF-induced reduction of sociability. The relationship between BDNF and the complement system has not previously been reported; nevertheless, complement C3 signaling starting at C1q is crucial for the experience-dependent synaptic pruning of microglia [2, 7]. Thus, decreased C1q and C3ar1 [71–74] expression may reduce microglial pruning and inhibit mPFC circuit purification. In addition, the relationship between the complement system and psychiatric disorders is gradually becoming more evident [75, 76], indicating that further investigations are needed. Our experiments with mice have a limitation. The duration during which MG-BDNF overexpression remained suppressed may be critical rather than the timing of the DOX administration initiation. However, we have shown that resocialization after P35 in j-SI mice does not improve either sociability or mPFC function [12, 16–18], which may support the timing specificity in the current study.

Childhood experiences were also associated with *BDNF* expression in human peripheral M2 macrophages in this study. Microglia and macrophages should be considered separately [48] as primitive myeloid progenitor cells (microglia’s origin) migrate from the yolk sac into the brain from the embryonic period [77]. Thus, results in mice and humans cannot be directly compared; however, a relationship exists between childhood experiences and *BDNF* expression in macrophages that share similarities in CD11b expression and phagocytic capacity with microglia [48, 49] (the resident macrophages in the brain [49, 50]). M1 macrophages have high antigen-presenting activity and pro-inflammatory cytokine-releasing capacity. In contrast, M2 macrophages have multiple roles aside from inflammation, including anti-inflammatory responses and tissue remodeling, and secrete numerous growth and neurotrophic factors [78–80]. M2 macrophages are also implicated in the pathobiology of neuropsychiatric and neurodegenerative disorders [81–83]. Whether high levels of *BDNF* in M2 macrophages are associated with reduced sociability remains unclear; however, BDNF abnormalities have been identified in humans with autism spectrum and posttraumatic stress disorders [84–86]. They may also be associated with the reduced sociability of these disorders [87, 88].

In conclusion, these findings indicate that MG-BDNF is critical in developing social behaviors and mPFC function in a time-specific manner, potentially related to juvenile social experience-dependent social development. Our results provide new insights into experience-dependent social behavior formation and mPFC development.

## Figures and Tables

**Table 1:** Correlation between human macrophage BDNF expression and adverse childhood experiences

	M1		M2	
CATS	rs	p value	rs	p value
Total score	0.069	0.675	0.409	0.010 *
Sub-item scores				
Sexual abuse	−0.114	0.488	0.293	0.071
Punishment	0.230	0.159	0.396	0.013 *
Neglect	0.008	0.960	0.348	0.030 *
Emotional abuse	−0.043	0.795	0.321	0.046
Others	0.093	0.574	0.367	0.022 *
